# Measures of statistical dispersion based on Entropy and Fisher information

**DOI:** 10.1186/1471-2202-12-S1-P255

**Published:** 2011-07-18

**Authors:** Lubomir Kostal, Petr Lansky, Ondrej Pokora

**Affiliations:** 1 Department of Computational Neuroscience. Institute of Physiology, Praha, Czech Republic

## 

We propose and discuss two information-based measures of statistical dispersion suitable to description of interspike interval data. The measures are compared with the standard deviation. Although the standard deviation is used routinely, we show that it is not well suited to quantify some aspects of dispersion which are often expected intuitively, such as the degree of randomness. The proposed dispersion measures are not mutually independent, however, each describes the firing regularity from a different point of view. We discuss relationships between the measures and describe their extreme values. We also apply the method to real experimental data from spontaneously active olfactory neurons of rats. Our results and conclusions are applicable to a wide range of situations where the distribution of a continuous positive random variable is of interest.
